# A revolutionary tool: CRISPR technology plays an important role in construction of intelligentized gene circuits

**DOI:** 10.1111/cpr.12552

**Published:** 2018-12-05

**Authors:** Qun Zhou, Hengji Zhan, Xinhui Liao, Lan Fang, Yuhan Liu, Haibiao Xie, Kang Yang, Qunjun Gao, Mengting Ding, Zhiming Cai, Weiren Huang, Yuchen Liu

**Affiliations:** ^1^ Department of Urology, Shenzhen Second People′s Hospital Clinical Medicine College of Anhui Medical University Shenzhen China; ^2^ Department of Urology the First Affiliated Hospital of Shenzhen University Shenzhen China

**Keywords:** artificial, CRISPR technology, gene circuits, gene editing, intelligentized

## Abstract

With the development of synthetic biology, synthetic gene circuits have shown great applied potential in medicine, biology, and as commodity chemicals. An ultimate challenge in the construction of gene circuits is the lack of effective, programmable, secure and sequence‐specific gene editing tools. The clustered regularly interspaced short palindromic repeat (CRISPR) system, a CRISPR‐associated RNA‐guided endonuclease Cas9 (CRISPR‐associated protein 9)‐targeted genome editing tool, has recently been applied in engineering gene circuits for its unique properties‐operability, high efficiency and programmability. The traditional single‐targeted therapy cannot effectively distinguish tumour cells from normal cells, and gene therapy for single targets has poor anti‐tumour effects, which severely limits the application of gene therapy. Currently, the design of gene circuits using tumour‐specific targets based on CRISPR/Cas systems provides a new way for precision cancer therapy. Hence, the application of intelligentized gene circuits based on CRISPR technology effectively guarantees the safety, efficiency and specificity of cancer therapy. Here, we assessed the use of synthetic gene circuits and if the CRISPR system could be used, especially artificial switch‐inducible Cas9, to more effectively target and treat tumour cells. Moreover, we also discussed recent advances, prospectives and underlying challenges in CRISPR‐based gene circuit development.

## 1 INTRODUCTION

Over the past decade, advances in synthetic biology and bioinformatics have been made in the design of reasonable and efficient artificial gene signalling circuits with desired functionality.^1^ Artificial gene circuits were first constructed in prokaryotic cells and gradually implemented in eukaryotic cells.^2^, ^3^, ^4^ Currently, signal networks within living organisms and cells can be reprogrammed to alter the original oncogenic signalling pathway.^5^

### 1.1 The concept and composition of artificial genetic circuits

With progress in human genome sequencing research, logical relationships between related genes and their products and clarification of gene expression regulation have advanced, but studies remain to be done.^6^ Simple genetic circuits consist of elements such as a promoter sequence, coding sequence, terminator, transcription factors and ribosome sites that are based on known cellular relationships,^7^ because all cells use their genetic circuitry ultimately to regulate their behaviour and their reactions to the environment.

Biological technology industries have undergone unprecedented changes in the field of precision tumour treatments. It is now possible to edit the genome through integration of its components and modules. Artificial genetic circuits can connect different genes to complete a specific biological function, and generally include three functional modules, sensors, processors and actuators.^8^ Sensors measure the cellular information and transmit this to the processors. The processors can be designed as switching signals using logic relationship analyses. Finally, actuators perform the procedures set by the command. Numerous genetic circuits have been developed, including logic gates,^9^ analogue computing circuits,^10^ counters and clocks,^11^ and memory devices.^12^

Compared with traditional single‐target gene therapy strategies, gene circuits use elements such as biosensor‐quantitative signal detection and comprehensive analysis of a variety of tumours, in order to accurately distinguish between cancer cells and normal cells. Therapeutically, the artificial tumour suppressor gene systems may be used to initiate cellular self‐destruction programs, which can specifically and efficiently kill tumour cells or inhibit their malignant biological behaviours. Therefore, in the future, artificial gene circuits may provide accurate targeted therapy of tumours.^13^, ^14^

### 1.2 CRISPR/Cas technology

Clustered regularly interspaced short palindromic repeat (CRISPR) sequences and their associated proteins (Cas) were first identified in the immune system of bacteria and archaea,^15^ as protective mechanisms from exogenous DNA. With recent advances in technology, the CRISPR system has been greatly improved, and its application has been continuously expanded. CRISPR technology provides a robust and multiplex genome editing tool, enabling researchers to precisely manipulate specific genomic elements, to facilitate the elucidation of target gene functions in cells and disease states. CRISPR/Cas technology has become a major tool in the field of biological medicine research,^16^ for gene editing,^17^ for regulating gene signal networks^18^, ^19^ and in synthetic biology research.^20^, ^21^

The CRISPR Cas9 system is composed of single‐guide RNA (sgRNA) and Cas9 protein with endonuclease activity. The crRNA (CRISPR RNA) maturation from pre‐crRNA requires a tracrRNA based on base pairing, which fuses with single‐chain guide RNA; RNAse III plays an important role in this process. According to the effectors of the Cas proteins, the CRISPR/Cas system is divided into two primary categories: class 1 (types I, III and IV), and class 2 systems (types II, V and VI).^22^, ^23^ The main characteristic of class 1 CRISPR‐associated protein is that multiple Cas protein subunits form complexes; in contrast, the class 2 Cas protein consists of a single effector protein.

**Figure 1:**
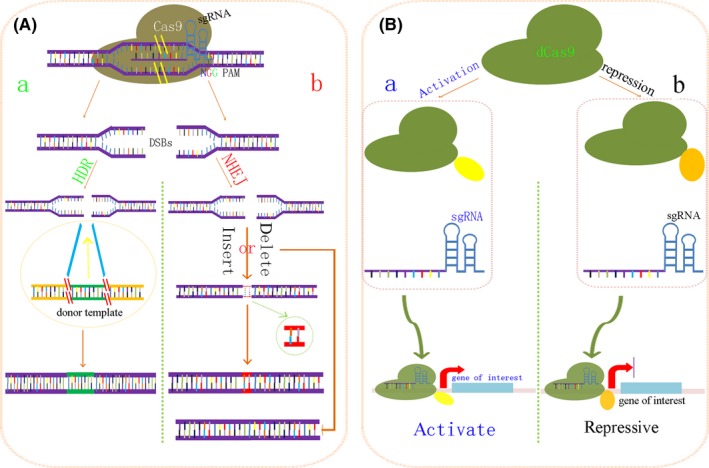
Schematic illustrations of the gene editing mediated by Cas9 or dCas9 proteins. PAM (protospacer adjacent motif). A, Double‐stranded breaks (DSBs) mediated by CRISPR Cas9 and the two repair pathway. (A) The donor template gene sequence is linked to the DNA double‐stranded breaks site by homologous recombinant arms. This pathway is called the homology‐directed repair pathway (HDR); (B) the two ends of the DNA sequence are directly connected by the ligase to repair the double‐stranded breaks site. This pathway is called the non‐homologous end‐joining pathway (NHEJ). B, dcas9‐mediated gene activation or inhibition. (A) dCas9 proteins promote transcriptional activation of downstream genes by fusing transcription activators, the yellow parts represent the transcription activation domain, such as VP64, P65, RTA and VPR (a fusion protein of VP64, P65 and RTA); (B) dCas9 inhibits downstream gene expression by fusing transcription inhibitors, the orange parts are the transcription inhibitor domains, such as Krüppel‐associated box (KRAB), etc

The type II CRISPR/Cas system from *Streptococcus pyogenes* (spCas9) has been extensively used in synthetic biology due to its high specificity, efficiency, versatility and safety. In the process of genome editing, Cas9 and sgRNA complex binding to the target DNA sequence is not random. Cas9 protein binds to a DNA target, recognizing a protospacer adjacent motif sequence (PAM, usually 5′‐NGG), and by sgRNA binding to the complimentary region adjacent of the PAM. Studies in eukaryotic cells and animal models have indicated that the CRISPR Cas9 system can efficiently cleave DNA, resulting in double‐stranded DNA breaks (DSBs), which then insert or delete target genes by a homology‐directed repair pathway (HDR) or a non‐homologous end‐joining pathway (NHEJ). Furthermore, a dead‐Cas9 (dCas9) was obtained by mutation of two sites, D10A and H841A, in the *Cas9* gene.^24^ The dCas9 can inhibit or activate the expression of target genes through the fusion expression of the functional domain of transcriptional inhibitors or activators.^25^, ^26^, ^27^, ^28^, ^29^ Figure 1 illustrates the process of gene editing by Cas9 protein.

## 2 ARTIFICIAL GENE CIRCUITRY BASED ON CRISPR/CAS TECHNOLOGY

**Figure 2:**
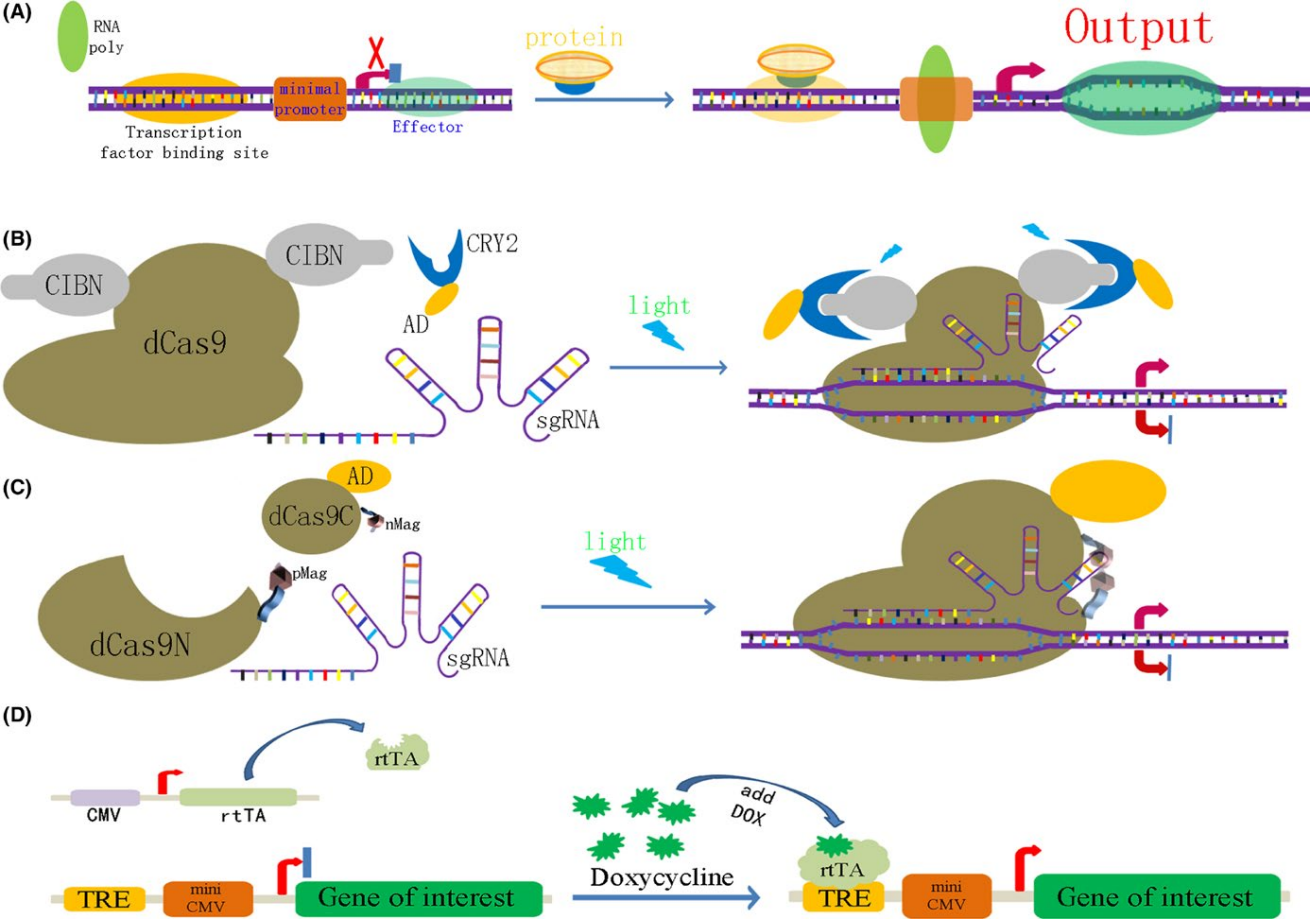
Schematic representation of the artificial gene circuitry based on CRISPR/Cas technology. A, Artificial sequences of transcription factor binding site were inserted upstream of the CRISPR Cas9 gene sequence to control gene expression by sensing intracellular signal proteins. In malignant tumour cells, some specific transcription factors are abnormally activated, such as β‐catenin and NF‐κB. The expression of downstream CRISPR/Cas genes is turned on when abnormal signal proteins in malignant cells bind to the transcription factor binding site and RNA polymerase (RNA poly) was recruited to TATA box. Effector means CRISPR Cas9 protein. B, Light‐inducible gene expression device in cancer cells based on CRISPR Cas9 technology. Blue light stimulation induces heterodimerization between *Arabidopsis thaliana* cryptochrome 2 (CRY2) and its binding partner CIBN (cryptochrome‐interacting basic helix‐loop‐helix protein 1). Therefore, the transcriptional activation domain (AD) fused with the CRY2 protein is carried to the specified region and promotes the expression of downstream genes. C, Schematic process of the light‐inducible CRISPR dCas9. The dCas9 is split into two fragments lacking nuclease activity, and the dCas9 fragments are fused with light‐inducible dimerization domains (pMag and nMag). Blue light stimulation induces heterodimerization between pMag and nMag, which enables split dCas9 fragments to reassociate, thereby reconstituting RNA‐guided nuclease activity. D, Schematic diagram of a small molecular artificial switch system. The combination of doxycycline and reverse tetracycline transcriptional activator (rtTA), results in changes in the conformation of rtTA, and the combination of activated rtTA and Tet‐responsive element (TRE) results in the expression of target genes, such as *Cas9* gene

CRISPR Cas9 technology provides a highly efficient tool for genetic circuit design, greatly improving the design and efficiency of each component. There have been attempts to use CRISPR technology to construct simple gene circuits, which can be used in gene therapy to treat tumours.^30^, ^31^ In addition, the application of CRISPR technology to build genetic circuits is not only limited to tumour cells but also can be used more widely in plants^32^, ^33^ and animals.^34^ However, the safety of artificial gene circuits in humans has been controversial, and it has therefore been a problem for scientists to reconstruct a safe and effective gene circuit by using genetic components. To solve this problem, artificial switches have been used in gene circuits to improve safety. Several CRISPR‐switchable cellular signalling pathways have been designed that are controlled by external signals, including endogenous proteins,^35^ light^36^, ^37^, ^38^ and small molecules^20^, ^39^ (Figure 2). The use of artificial, switchable systems promoted the safety of gene therapy, but the devices that perceive internal signal proteins will be the next generation of gene therapy tools.

### 2.1 CRISPR dcas9‐mediated signal conductors

In recent years, several artificial synthetic elements have been developed to regulate cellular gene signal networks. Nissim et al^40^ constructed a dual‐promoter integrator to differentiate colon cancer cells from normal fibroblasts cells. Xie et al^41^, ^42^, ^43^ successfully developed a specific cell classifier to effectively distinguish HeLa cells from other types of cells by detecting the differential expression of five endogenous miRNA molecules. Morsut et al^44^ constructed dual‐receptor AND‐gate T cells according to their tumour‐associated antigen‐receptor system. This engineered T cells were designed two synthetic notch receptors on the cell surface, called dual‐receptor, enabling T cells to recognize two tumour‐specific antigens, which were activated only when tumour cells expressed both antigens at the same time. The route to Boolean logic improves the ability to specifically identify tumour cells. In the process of tumour cells development, endogenous proteins are directly involved in the regulation of internal signal networks, and may play more important roles in promoting the tumour than miRNAs and antigens. It may therefore be more effective to exploit the proteins that regulate the key signalling pathways.

**Figure 3:**
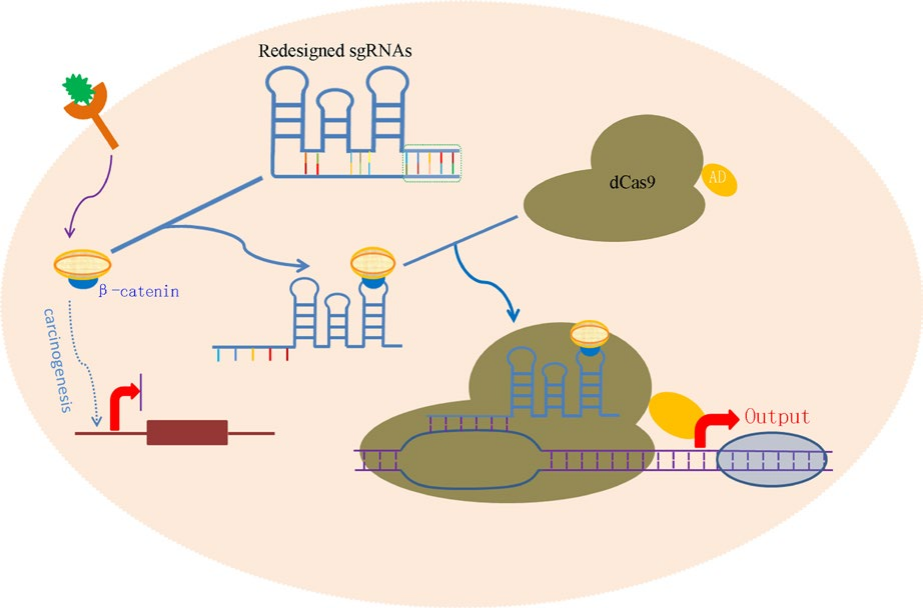
Schematic illustrations of the signal conductor that links one signal with another based on CRISPR/Cas technology. The β‐catenin activates the Wnt pathway and promotes the proliferation of tumour cells. The redesigned sgRNA preferentially binds to endogenous β‐catenin and then the redesigned sgRNA is activated. Then, the redesigned sgRNA coupled with dCas9 protein binds to the target sequence and activates the Output gene, such as endogenous tumour suppressor gene (eg, *p53*) or apoptosis gene (eg, *p21* and *caspase 3*), enabling the tumour cells to redirect oncogenic signalling to an anti‐oncogenic pathway

A critical obstacle to creating complex genetic circuits is the lack of engineered gene regulatory elements, and CRISPR technology provides an efficient tool to greatly improve the design of genetic circuits. Liu et al^45^, ^46^ developed CRISPR‐mediated signal conductors to recognize multiple protein signals at the same time, which overcame the deficiency of the traditional genetic circuits. The study creatively integrated the RNA riboswitch into the 3' end of the sgRNA to construct a novel riboswitch that controlled the sgRNA. The RNA riboswitch consisted of an aptamer and double‐stranded antisense RNA, and a double‐chain antisense arm that closed without a specific signal. Antisense arms can be opened through the strand displacement reaction when the aptamer perceives inner signal protein changes. The opened antisense arms target DNA sequences and play a role in transcriptional activation or inhibition of malignant phenotypes of tumour cells (Figure 3). CRISPR dcas9 transcription factors (CRISPR‐TFs) regulated by protein signals can dynamically respond and effectively process different tumour signals, change the direction of cell information flow, and could become important tools for constructing artificial gene circuits. Therefore, a novel CRISPR dCas9‐mediated signal conductor may have many applications in synthetic biology and cancer therapy.

### 2.2 Simultaneous multi‐gene regulation based on CRISPR Cas9 technology

Compared with traditional gene editing tools, such as transcription activator‐like effector nucleases (TALENs) and zinc‐finger nucleases (ZFNs), CRISPR Cas9 technology is not only efficient and convenient but also has an ability to regulate multiple targets simultaneously within single cell.^47^ The dCas9 protein fused with transcriptional repressing or transcriptional activating domains were used to downregulate or upregulate specific single genes. What's more, with the maturity of CRISPR technology, the multi‐gene targeted tandem sgRNA array was also designed to regulate the expression of multiple genes simultaneously.^25^ However, genes always play an important role in the growth and development of tumour cells, but the function of genes is complex and volatile, therefore, different regulatory modalities need to be chosen for different genes.

The exciting advantage of CRISPR/Cas is its ability to control multiple endogenous genes with optional ways within a single cell. It provides only one regulatory method when dCas9 directly fused with one transcription‐related domain (ie, activation or repression). However, previous studies^29^ have shown that sgRNA can recruit different transcription‐related domains and thus has the ability to upregulate and downregulate different genes simultaneously. RNA‐binding proteins are used to carry the corresponding transcription‐related domain and specifically bind to the RNA hairpin structures. After the fusion of different RNA hairpin structures and sgRNA, the corresponding genes can be transcriptionally activated or suppressed. This technology has provided the prospect of broad application and great application value for precise treatment of cancer cells, and has provided more possibilities for biomedical science.

### 2.3 Engineering cell signalling based on the novel CRISPR dCpf1 technology

Although CRISPR Cas9 technology has played a significant role in the precise treatment of tumours, the method of delivery to the target cell remains a problem. The size of the spCas9 gene is about 4.2 kb, which is the most widely used in biology. However, the process of expressing Cas9 protein in cells also requires regulatory elements, such as promoters. In addition, the expressed Cas9/dCas9 protein in cells alone does not perform its biological function, and it is also required to be co‐expressed in cells as other components (such as sgRNA, donor template, transcriptional activator or transcriptional repressor). The size of co‐expression vector is larger than the existing virus carrier capacity; therefore, it is difficult to load into an adeno‐associated virus vector (AAV). Finding a novel delivery method is of great importance for in vivo therapeutic application. Therefore, it is necessary to exploit a new CRISPR toolkit that efficiently edits the gene.

At present, the current studies still use a miniaturized or split spCas9 protein and CRISPR system. The initial scheme exploited a CRISPR/Cas protein with the small size, such as a small size Cas9 from*Staphylococcus aureus* (SaCas9)^48^ (3.2 kb) and a newfound genome editing tool CasX^49^ (3.0 kb), or constructed a mini‐spCas9 system.^50^, ^51^, ^52^ However, the gene editing efficiency of miniaturized CRISPR tools may be far less than that of wild‐type spCas9 and CRISPR from Prevotella and Francisella 1 (Cpf1, also named Cas12a). Recently, a novel gene editing system has been developed for eukaryotic cells based on CRISPR dCpf1.^53^ Cpf1 is a smaller protein than Cas9, and it may be easily packaged for delivery. The dead Cpf1 (dCpf1) is produced by mutation of Cpf1, which has great application value to signal amplification and transcriptional regulation of genome editing. It is also possible to construct a CRISPR‐AAV integrated system by optimizing the relevant regulatory elements of dCpf1. This would greatly improve the application value of intelligentized gene circuits in future accurate cancer therapy.

## 3 THE APPLICATION OF GENE CIRCUITS IN GENE THERAPY FOR CANCER CELLS

The occurrence of cancer is closely related to the disorder of gene expression and regulation in cells, and previous gene therapy of tumour cells is usually targeted at single‐gene intervention to improve the malignant phenotype of cancer cells or kill cancer cells. With the intense research of human gene sequencing, a large amount of data has been obtained in a short time through the integration of biological information of gene expression, which reveals the logical relationship between related genes and their products, and clarifies that gene expression regulation is neither isolated nor single, but mutually restricted and interrelated.^6^ The occurrence and development of cancer cells is a complex process, which involves complex information networks interacting with many signalling molecules.^54^ Therefore, regulation and intervention of cellular signalling pathways is usually complex and difficult, which involve dynamic perception and multilayer regulation.

Synthetic gene circuits enable cells to perceive cellular signal proteins, evaluate messages, and have programmable behaviours that respond dynamically to inputs. It has become popular to construct gene circuits in tumour cells by using synthetic biology. Nissim et al^55^ first tested the possibility for constructing gene circuits based on the CRISPR Cas9 system. They used an integrated RNA and CRISPR/Cas toolkit to achieve multiplexed and programmable regulation of gene networks in human HEK‐293T cells. Similar works have also been reported by the other two teams, and both concluded that CRISPR Cas9 transcription factors can be used to construct synthetic gene circuits in human cells.^39^, ^56^ After that, our group showed a synthesized AND‐gate genetic circuits based on CRISPPR Cas9 for identification of bladder cancer cells in vitro.^57^ By extending sgRNA to include modified riboswitches that recognize oncogenic signals, we create the CRISPR Cas9‐based “signal conductors” that redirected oncogenic signal transduction by controlling simultaneous bidirectional (ON‐OFF) gene transcriptions. The CRISPR signal conductors also reduced tumour growth in a mouse model. Ma et al^43^ constructed logic AND circuits by integrating multiple split dCas9 domains, which were useful to reduce the size of synthetic circuits. This circuit could be used to identify HeLa cancer cells. Overall, such CRISPR Cas9 circuits will be particularly useful in biomedical applications.

Although current studies have shown that gene circuits based on CRISPR/Cas technology can intelligently modify intracellular signal networks, regulate gene expression from the DNA level and inhibit the growth and development of tumour cells, the clinical applications are still not currently available.

## 4 CHALLENGES AND LIMITATIONS OF THE GENE CIRCUITS BASED ON CRISPR/CAS9 TECHNOLOGY

### 4.1 The limitations of gene circuit technology

The application of gene circuits is of great significance for the precise treatment of tumours. However, there are still many deficiencies in this technology, as well as several crucial problems and technical challenges that must be solved. First, the development of tumours is a complex process involving complicated information networks that interact with multiple signalling molecules.^20^ Use of a single specific signalling molecule or oncogene may not effectively distinguish between normal and tumour cells; therefore, single‐targeted chemotherapy drugs and gene therapy used in current clinical treatments have poor effects and significant side effects, and this limits the application of gene therapy in cancer cells. Second, in the construction of gene circuits, it is difficult to build an artificial gene circuit that cooperatively works in cells without crosstalk. How to design such functional elements has become a basic challenge for the synthetic gene circuit field.^58^ Therefore, it is important to correct the insufficiencies of the currently used gene circuits.

### 4.2 The challenge of CRISPR Cas9 technology

It is usually fused with transcriptional activators (VPR) or transcriptional repressors (KRAB) to enhance the function when dCas9 protein exerts its transcription activation or transcription inhibition. The size of dCas9‐VPR/KRAB is larger than the existing virus carrier capacity; therefore, it is a difficult problem to load into an AAV. Exploitation of novel delivery technologies is of great importance for in vivo therapeutic applications. Despite the CRISPR/Cas system being a crucial tool in the field of synthetic biology, a high efficiency delivery pathway is also a major problem that should be addressed before use in clinical therapy. At present, the major carrier methods of delivery are through viral and non‐viral vectors, and among them, non‐viral vectors are safer for humans use, but still have limitations. First, the stability of the non‐viral vectors is not always predictable. It is known that components of the CRISPR dCas9 system remain stable until they reach the target binding sites. Second, non‐viral vectors have lower delivery efficiency compared to that of the viral vectors. These shortcomings have led to a significant reduction in the practical application of CRISPR dCas9 systems. However, AAVs have been used to treat diseases in clinics for many years, with good delivery efficiency and relatively high safety.^59^ AAV exists as a stable adjunct that does not integrate into the host cell's genome. Over the past 3 years, the ability of AAVs to carry out gene transfers in animals, including human target tissues, such as liver, retina, heart, muscle and central nervous system, has been proven.^60^, ^61^ Therefore, AAVs are still useful vectors for gene therapy for malignant tumour cells. Third, as an exogenous protein, Cas9 has safety and efficiency problems for use in humans. Doudna et al^62^, ^63^, ^64^ reduced the off‐target effects by exploiting a novel anti‐CRISPR technology, including a switch that made the CRISPR Cas9 complete its task and then lose its function. CRISPR Cas9 technology currently remains one of the most powerful genome editing tools, but its limitations are not to be ignored and should be addressed in the future.

## 5 CONCLUSIONS AND PROSPECTS

With the development of genome editing, gene circuit technology has become more precise and demanding.^65^, ^66^ We conducted a systematic review of gene circuit development based on CRISPR technology and summarized the applications of gene circuits in tumour treatments. Based on existing studies, the potential application value of intelligentized gene circuits in the field of tumour gene therapy was proposed. There are limitations in traditional gene circuit technology, including the ability to specifically differentiate tumour cells from normal cells. Scientists have developed a series of efficient and targeted transcription factor components based on CRISPR technology. These novel designs extend the toolbox of gene editing, and enable the construction of intelligentized gene circuits, such as logic gates,^9^ signal conductors,^45^ analogue computing circuits,^10^ counters and memory devices.^11^, ^12^

Despite CRISPR technology being widely used in synthetic biology and biomedicine, there are still limitations. The off‐target effects remain a major concern, and scientists have been working on various ways to solve this problem.^19^, ^67^, ^68^ For example, three neutral amino acids (alanine) were used to replace positively charged amino acids (K848A/K1003A/R1060A or K810A/K1003A/R1060A) at their corresponding positions in the dCas9 protein, in order to reduce the non‐specific binding of dCas9 to DNA (with a negative charge).^69^ In addition, a truncated sgRNA with only 17‐19 nucleotides was constructed for targeted DNA sequences.^70^ Another major problem is that CRISPR systems are difficult to package using AAVs. Therefore, novel transcriptional activators (TFs) and transcriptional inhibitors were used, such as the dCpf1 system, a miniaturized CRISPR dCas9 system. With the development of these technologies, the CRISPR/Cas system may become more sophisticated and efficient for use in the future.

In conclusion, with the continuous development of genome editing and gene circuit technology, the abovementioned deficiencies will eventually be resolved. This will provide a method for its application in clinical treatment. Intelligentized gene circuits based on CRISPR technology will be an important research direction in the field of tumour gene therapy in the future.

## CONFLICT OF INTEREST

All authors declare that there is no conflict of interest.
